# Portfolios in Postgraduate Medical Training: a Formative or Summative Tool?

**DOI:** 10.15694/mep.2019.000068.1

**Published:** 2019-03-26

**Authors:** Claire Wilson

**Affiliations:** 1 King's College London

**Keywords:** formative assessment, summative assessment, portfolios

## Abstract

This article was migrated. The article was marked as recommended.

The recent case in the UK of trainee paediatrician Hadiza Bawa-Garba shone a light on the widespread uncertainty among trainees about how to engage with reflective portfolios in their ongoing professional development. Here I discuss the dilemma within a formative versus summative assessment framework and argue that greater clarity is required on the intended purpose of portfolios.

## Portfolios in Postgraduate Medical Training: a Formative or Summative Tool?

The recent fitness to practice case in the UK of Hadiza Bawa-Garba, whose reflections documented in her e-portfolio were used as evidence against her during the case, has called into question the role of portfolios in UK postgraduate medical training ( Dyer and Cohen, 2018). As a psychiatry trainee, I have reflected on how I use my portfolio in my training. Should I be using it as a formative tool for personal development and reflect openly within it, or should I be viewing it as a summative tool which could also be used in a future fitness to practice case?

Schuwirth and van der Vleuten asserted that ‘if you do not know what you are assessing it also becomes very difficult to know how you can best assess.’( Schuwirth and Van der Vleuten, 2011) What is the aim of portfolios? Is it to measure competence within a framework of clearly defined outcomes or is it to assess professionalism more broadly, facilitating feedback and reflection? Some have argued that explicit performance criteria are necessary to ensure robust and transparent professional regulation ( Crossley, Humphris and Jolly, 2002). However, this approach assumes that performance criteria can be defined and has been described as ‘reductionism’ by those who argue for a more holistic approach beyond the testing of individual curricular components ( Schuwirth and Ash, 2013). Moreover, there is evidence that feedback is best received within a formative context ( Gosling, 2002) and that this facilitates critical reflection ( Mann, Gordon and MacLeod, 2009).

Thus perhaps one of the many learning points from Bawa-Garba’s case is that it has highlighted the need for further evaluation of the role of portfolios in facilitating the development of trainees. I would argue that critical reflection and honest feedback is likely best achieved via formative assessment, whereas attainment of a common standard is likely best evidenced within a summative assessment. However, there is an urgent need to provide trainees with clarity on their intended purpose in order to best engage us in their use as part of our training.

## Take Home Messages

Greater clarity is required on the role of portfolios within medical training, whether it be formative or summative in order to enable trainees to better engage in ongoing professional development practices.

## Notes On Contributors

Dr Claire Wilson, BSc(Hons) MBChB MRCPsych, is an MRC Clinical Research Training Fellow currently conducting her PhD research in the Section of Women’s Mental Health at the Institute of Psychiatry, Psychology and Neuroscience, King’s College London. She is also a specialist registrar in psychiatry at South London and Maudsley NHS Foundation Trust. She has a keen interest in medical education and is currently undertaking a Diploma in Clinical Education at King’s College London, having recently obtained a PGCert with distinction.
