# Supplementary material for: Portfolios in Postgraduate Medical Training: a Formative or Summative Tool?
Source: MedEdPublish (2016). 2019 Mar 26;8:68. [Version 1] doi: 10.15694/mep.2019.000068.1 (PMC10712585; doi:10.15694/mep.2019.000068.1)
Supplement: Peer review history

# Reviewer report: Reviewer response for version 1

**DOI:** 10.21956/mep.19797.r29774

**Published:** 2019-03-30

**Authors:** sathyanarayanan varadarajan

**Affiliations:** 1 SRM Medical College, SRM University

This review has been migrated. The reviewer awarded 4 stars out of 5 This article has appeared in the category of personal view / opinion piece, in which the author has raised a pertinent question on Portfolios in Postgraduate Medical Training: a Formative or Summative Tool? , based on the recent case in UK of a trainee pediatrician, which has created uncertainty among trainees on how to engage reflective portfolios.In this article, the author has discussed the dilemma within a formative versus summative assessment framework by quoting several authors and argues that greater clarity is required on the intended purpose of portfolios.This article is very much relevant to medical educators who are involved in Postgraduate medical training and need further elaborate research into this question.

Reviewer Expertise:

NA

No decision status is available

# Reviewer report: Reviewer response for version 1

**DOI:** 10.21956/mep.19797.r29773

**Published:** 2019-03-26

**Authors:** Leila Niemi-Murola

**Affiliations:** 1 University of Helsinki

This review has been migrated. The reviewer awarded 3 stars out of 5 Thank you for the opportunity to review this interesting letter. As an outsider, I am not familiar with the case of Dr Bawa-Garba. However, the author comments on a very important issue related to portfolios. In some countries residents document their progress in portfolios. Critical reflection is an important part of learning. Both the resident and the supervisor have access to the portfolio, which is basically confidential. In other words, the portfolio is primarily for formative and secondarily for summative assessment. The author has a point - if there is a risk that the reflective portfolio will be used in order to assess the resident's fitness for practice, we will lose the reflectice component vital for professional development.

Reviewer Expertise:

NA

No decision status is available
